# Willingness-to-pay for a hypothetical Ebola vaccine in Indonesia: A cross-sectional study in Aceh

**DOI:** 10.12688/f1000research.20144.2

**Published:** 2020-07-13

**Authors:** Mudatsir Mudatsir, Samsul Anwar, Jonny K. Fajar, Amanda Yufika, Muhammad N. Ferdian, Salwiyadi Salwiyadi, Aga S. Imanda, Roully Azhars, Darul Ilham, Arya U. Timur, Juwita Sahputri, Ricky Yordani, Setia Pramana, Yogambigai Rajamoorthy, Abram L. Wagner, Kurnia F. Jamil, Harapan Harapan

**Affiliations:** 1 Medical Research Unit, School of Medicine, Universitas Syiah Kuala, Banda Aceh, Indonesia; 2 Tropical Diseases Centre, School of Medicine, Universitas Syiah Kuala, Banda Aceh, Indonesia; 3 Department of Microbiology, School of Medicine, Universitas Syiah Kuala, Bnada Aceh, Indonesia; 4 Department of Statistics, Faculty of Mathematics and Natural Sciences, Universitas Syiah Kuala, Banda Aceh, Indonesia; 5 Department of Family Medicine, School of Medicine, Universitas Syiah Kuala, Banda Aceh, Indonesia; 6 Department of Microbiology, Faculty of Medicine, Malikussaleh University, Lhokseumawe, Indonesia; 7 Institute of Statistics, Jakarta, Indonesia; 8 Department of Economics, Faculty of Accountancy and Management, Universiti Tunku Abdul Rahman, Selangor, Malaysia; 9 Department of Epidemiology, Department of Epidemiology, University of Michigan, Ann Arbor, USA; 10 Department of Internal Medicine, School of Medicine, Universitas Syiah Kuala, Banda Aceh, Indonesia

**Keywords:** Ebola vaccine, Ebola virus disease, Indonesia, vaccine acceptance, willingness-to-pay

## Abstract

**Background:** Some Ebola vaccines have been developed and tested in phase III clinical trials. However, assessment of whether public have willingness to purchase or not, especially in unaffected areas, is lacking. The aim of this study was to determine willingness to pay (WTP) for a hypothetical Ebola vaccine in Indonesia.

**Methods:** A cross-sectional study was conducted from 1 August to 30 December 2015 in five cities in Aceh province of Indonesia. Patients’ family members who visited outpatient departments were approached and interviewed about their sociodemographic characteristics, knowledge of Ebola, attitude towards vaccination practice and their WTP for a hypothetical Ebola vaccine. A multivariable linear regression model assessed the relationship between these explanatory variables and WTP.

**Results: **During the study, 500 participants were approached and interviewed. There were 424 (84.8%) respondents who completed the interview and 74% (311/424) expressed their acceptance for an Ebola vaccine. There were 288 participants who were willing to pay for an Ebola vaccine (92.6% out of 311). The mean of WTP was US$2.08 (95% CI: 1.75-2.42). The final multivariable model indicated that young age, high educational attainment, working as a private employee, entrepreneur or civil servant (compared to farmers), being unmarried, and residing in a suburb (compared to a city) were associated with higher WTP.

**Conclusions: **Although the proportion of the participants who would accept the Ebola vaccine was relatively high, the amount they were willing to pay for Ebola vaccine was very low. This finding would indicate the need of subsidies for Ebola vaccine in the country.

## Introduction

Ebola virus disease (EVD), formerly called as Ebola hemorrhagic fever, is a disease characterized by high mortality in human populations ^1^. EVD is caused by Ebola virus (EBOV) which is an enveloped, filamentous, and non-segmented negative-strand RNA virus ^2^. EBOV first emerged in tropical areas of Africa – in the countries now known as the Democratic Republic of the Congo and South Sudan – in 1976, and was recognized as a new viral hemorrhagic fever ^3^. Since then, EVD outbreaks have been reported intermittently. Recently there was an outbreak of almost 28,610 cases and 11,308 deaths, mostly affecting West Africa, but also spreading to Europe, North America, and Asia ^4, 5^. In Asia, EVD cases were reported in the Philippines ^6^. Although no cases have yet been reported in Indonesia, many travelers pass through the country. Since 2017, several outbreaks of EVD have impacted the Democratic Republic of the Congo, with over 2,000 reported cases as of June 2019 ^7^.

EVD is a highly fatal disease and can be economically burdensome in affected countries. The case fatality rate of EVD ranges from 25% to 100%, with an average of approximately 68% ^8, 9^. The highest rates of mortality are in infants and children ^10^. It is estimated that between $2.8 and $32.6 billion was spent to control the EVD outbreaks of 2014–2016 ^11^. Accordingly, the World Health Organization (WHO) declared EVD outbreak as a Public Health Emergency of International Concern with severe global economic burden in August 2014 ^12^. There is no specific treatment for EVD beyond supportive care.

Development of a safe and effective Ebola vaccine is a key component to future programs to control EVD ^13^. Several vaccines have been developed and tested in phase III clinical trials, such as rVSV-EBOV and the combination of Ad26-ZEBOV and MVA-BN Filo ^14^. The trials demonstrated that these vaccines have good effectiveness and provide robust protection against EVD; no EVD case have been reported among vaccinated individuals ^14^. Vaccine development will be beneficial for people living in West Africa and other regions affected by Ebola outbreaks. The vaccine has had some use in the current outbreaks in the Democratic Republic of the Congo ^15^. However, the problem with any new vaccine, particularly vaccines that require payment, is the public response, and whether members of the general population are willing to purchase the vaccine. A previous study reported high willingness to pay (WTP) for an Ebola vaccine in West Africa ^16^. However, in areas not yet affected, the results might differ because community members might lower perceptions of risk. This present study therefore aimed to investigate WTP for Ebola vaccine in Indonesia, a currently unaffected EVD country.

## Methods

### Study design and setting

Approximately 16 months after the Ministry of Health of the Republic of Indonesia raised an alert for EVD in Indonesia, a cross-sectional study was conducted to assess acceptance and WTP for a hypothetical Ebola vaccine among family members of patients with any illness admitted to eight health facilities (hospitals or Community Health Centres [ *Puskesmas*]) in four regencies (Nagan Raya, Aceh Selatan, Langsa and Banda Aceh) of Aceh province from 1 August to 30 December 2015. The study was conducted in. Aceh is located in the westernmost part of the Indonesian archipelago with a total population approximately 4,906,800 in 2014 ^17^.

### Study participants, sampling and sample size

Study participants were patients’ family members who visited infection and non-infection outpatient departments. Based on the population size of Aceh in 2014, the minimum sample size required was 385 ^18^. To recruit the samples, four regencies were selected randomly, and both urban and suburban areas were included. The number of participants from each study site was gathered proportionally to the size of regency’s population. To avoid repetitive field visits and to minimize the study design effect, the number of participants was increased for each study site. Family members who had resided in the specified regency for more than 3 months, were ≥17 years old, and were able to communicate in Bahasa Indonesia (the national language) were considered to be eligible for inclusion.

### Study instrument

To facilitate the interviews a set of a structured questionnaire, adapted from previous studies ^19, 20^, was used. Prior to use in the actual study, a pilot study was conducted to measure reliability of questionnaires among 25 participants in Lhokseumawe regency. For this pilot study, a Cronbach’s alpha score of ≥0.7 was considered good internal consistency. Edits were made to the questionnaire based on findings from the pilot study; the questionnaire is available in Indonesian and English as *Extended data* ^21^.

### Study variables

***Response variable.*** The response variable was WTP for a hypothetical Ebola vaccine. Prior to assessing their WTP, participants were provided with an introduction to the Ebola disease including the symptoms and modes of transmission. They were also informed of the following points: (a) infected patients need to be isolated and health care workers need to use special protection equipment while providing healthcare to the patients; (b) currently there is no available treatment for EVD; (c) the mortality rate of EVD is up to 90%; and (d) an Ebola vaccine would be safe and protective against EVD.

To assess the amount of money that participants would be willing to pay for a hypothetical Ebola vaccine, participants were asked whether they would be willing to pay for the vaccine using a list of Ebola vaccine prices: Indonesian Rupiah (IDR) 5.000, 10,000, 17.500, 37.500, 87.500, 150.000, and 300.000 (equivalent to US$ 0.37, 1.29, 2.78, 4.63, 6.48, 11.12, and 22.24). The possible responses were “very likely”, “likely”, “undecided”, “unlikely” or “very unlikely”. The WTP was defined as the highest price the participants said they were still “very likely” or “likely” willing to pay.

***Explanatory variables.*** *Sociodemographic data*: Sociodemographic factors such as age, gender, educational attainment, type of occupation, marital status, monthly income and urbanicity were collected from participants. The date of birth was recorded, converted into actual age and then collapsed into three groups. Educational attainment, defined as the highest level of formal education completed by respondents, was grouped into four groups. Participants were grouped into five types of occupation: (a) farmer; (b) private sector employee; (c) housewife; (d) entrepreneur (owned a small-scale business, or traders in a market); and (e) civil servant. Monthly income was grouped into: (a) less than 1 million Indonesian Rupiah (IDR) (equivalent to US$ 74.1); (b) 1 –2 million IDR (equivalent to US$ 74.1 - US$ 148.2); and (c) more than 2 million IDR (equivalent to US$ 148.2). Urbanicity included cities and suburbs.

*Socioeconomic status (SES):* SES was assessed based on 15 household assets owned by participants such as radio, landline phone, refrigerator, motorcycles, car, other electronics and house characteristics. Details of the full list of the household assets have been published previously ^20, 22^. The ownership of those assets was used to construct an asset index based on principal component analysis ^23^. SES classified into three tertiles, with the 1 ^st^ tertile the poorest and the 3 ^rd^ tertile the wealthiest.

*Attitude towards vaccination practice:* To measure attitude towards vaccination practice, five questions adopted from a previous study ^20^ was used. The questions included the attitude towards the safety and importance of vaccines, and previous experiences regarding vaccination practices. Participants responded to each statement on a five-point Likert-like scale ranging from “1=strongly disagree” to “5=strongly agree” with a higher score indicating a more positive attitude. The summed scores for this domain ranged from 5 to 25. Participants were classified as having a ”good” or ”poor” attitude based on a 75% cut-off point of the maximum score achieved by participants.

*Knowledge regarding Ebola:* To assess knowledge regarding Ebola, a set of six questions on transmission and prevention methods of EVD, adapted from a previous study ^19^, was used. Each valid response was given a score of one, whereas an incorrect response was given a score of zero. The summed scores for this domain ranged from 0 to 6, and knowledge of each participant was also classified into ”good” or ”poor” based on a 75% cut-off point.

### Data analysis

To assess the relationship between explanatory variables and WTP, a multivariable linear regression model was employed ^24, 25^. Various diagnostic assessments were used to check how well the data met the assumptions of linear regression. The variance inflation factor (VIF) ^26^, Glejser test ^27^ and Kolmogorov-Smirnov test ^28^ were employed to assess multicollinearity, heteroscedasticity and residual normality of the data, respectively. A VIF value of lower than 10 was used to define no multicollinearity between variables. A *P*-value greater than 0.05 in the Glejser test, and Kolmogorov-Smirnov test was applied to indicate no heteroscedasticity, and normal distribution of residuals, respectively ^24^.

Initial assessment indicated that the data violated all three assumptions and WTP values were then transformed using a natural logarithm function (Ln). After transformation, data showed better adherence to assumptions and therefore the transformed WTP values were used in linear regression model. In the initial multivariable model, all explanatory variables were included. Then, all explanatory variables that had *P* > 0.25 in this model were excluded from final linear regression model ^29^. Significance in the final model was assessed at an alpha level of 0.05. All associations between an explanatory factor and WTP were interpreted in relation to a reference category.

Because the outcome had been log-transformed, the mean estimated WTP in US$ and its 95% CI were calculated as $\text{Exp}\;(\text{X}\hat{\beta }\;+\;{\hat{\sigma }}^{2}/2)$ where $\hat{\beta }$ was the estimated regression coefficients (B) and $\;{\hat{\sigma }}^{2}$ was the mean squared error (MSE) of the multivariate model ^24, 30, 31^. All analyses were performed using SPSS (version 15, Chicago, USA). 

### Ethical approval

The protocol of this study was approved by Institutional Review Board of the School of Medicine, Universitas Syiah Kuala, Banda Aceh, Indonesia (Approval 315/KE/FK/2015). Prior to enrolment, the aims of the study were explained to the participants and they signed written consent forms. Participation in this study was voluntary and participants received no financial compensation.

## Results

The raw data for this study are available as *Underlying data* on Figshare ^32^.

### Participants’ characteristics

In this study, 500 participants were approached, all agreed to participate, but 76 were excluded due to incomplete data. Among those with completed data (424 or 84.8%), approximately 74% (311/424) of participants would accept an Ebola vaccine. There were 288 participants (92.6%, 288/311, of those who would accept an Ebola vaccine, or 67.9%, 288/424, of all participants with completed data), willing to pay for Ebola vaccine. The characteristics of the participants who were willing to pay for Ebola vaccine are presented in Table 1.

**Table 1.** Unadjusted relationship between sociodemographic factors and willingness-to-pay for a hypothetical Ebola vaccine (N=288).

Parameter	N (%)	Unstandardized coefficients				US$ estimate			*P*-value
		B	95% CI of B		SE	Mean	95% CI		
			Lower	Upper			Lower	Upper	
Intercept		1.469	0.864	2.073	0.307	5.915	4.368	7.461	<0.001
Age group (year)									
17–29 *(Ref)*	68 (23.6)								
30–44	147 (51.0)	-0.304	-0.556	-0.052	0.128	1.005	-0.542	2.551	0.018
≥45	73 (25.3)	-0.280	-0.581	0.022	0.153	1.029	-0.517	2.576	0.069
Gender									
Male *(Ref)*	137 (47.6)								
Female	151 (52.4)	0.110	-0.126	0.346	0.120	1.520	-0.027	3.066	0.359
Education									
Less than junior high school *(Ref)*	70 (24.3)								
Senior high school	136 (47.2)	0.300	0.043	0.557	0.130	1.837	0.291	3.384	0.022
Diploma	42 (14.6)	0.042	-0.372	0.456	0.210	1.420	-0.127	2.966	0.842
Graduated	40 (13.9)	0.526	0.042	1.010	0.246	2.304	0.757	3.851	0.033
Occupation									
Farmer *(Ref)*	77 (26.7)								
Private employee	24 (8.3)	0.741	0.267	1.215	0.241	2.857	1.310	4.403	0.002
Housewife	67 (23.3)	0.045	-0.284	0.375	0.167	1.424	-0.122	2.971	0.788
Entrepreneur	72 (25.0)	0.400	0.106	0.694	0.149	2.032	0.485	3.578	0.008
Civil servant	48 (16.7)	0.606	0.146	1.065	0.233	2.495	0.948	4.042	0.010
Marital status									
Unmarried *(Ref)*	10 (3.5)								
Married	278 (96.5)	-0.896	-1.438	-0.355	0.275	0.556	-0.991	2.102	0.001
Monthly income (IDR)									
<1 million *(Ref)*	127 (44.1)								
1 to ≤ 2 million	96 (33.3)	-0.107	-0.336	0.123	0.117	1.224	-0.323	2.770	0.360
>2 million	65 (22.6)	0.054	-0.315	0.424	0.188	1.437	-0.110	2.984	0.774
Urbanicity									
Suburb *(Ref)*	247 (85.8)								
City	41 (14.2)	-0.491	-0.842	-0.141	0.178	0.833	-0.714	2.379	0.006
Socio-economic status									
1 ^st^ tertile (Poorest) *(Ref)*	95 (33.0)								
2 ^nd^ tertile	96 (33.3)	-0.150	-0.418	0.119	0.136	1.172	-0.374	2.719	0.273
3 ^rd^ tertile (Wealthiest)	97 (33.7)	-0.076	-0.404	0.252	0.167	1.262	-0.285	2.808	0.649
Attitude towards vaccination practice									
Poor *(Ref)*	138 (47.9)								
Good	150 (52.1)	-0.029	-0.251	0.193	0.113	1.322	-0.224	2.869	0.797
Knowledge of Ebola									
Poor *(Ref)*	196 (68.1)								
Good	92 (31.9)	-0.201	-0.461	0.059	0.132	1.113	-0.433	2.660	0.129
Mean squared error (MSE)		0.617							
F value		4.281 (P<0.001)							
R ^2^		0.223							

CI, confidence interval; IDR, Indonesia rupiah; US$, American dollar; SE, standard error; *Ref*, reference group.

More than half (51.0%) of those who willing to pay for the vaccine were aged 30–44 years old, and 52.4% were female. A majority (75.7%) of them had finished their senior high school (year 12) and none of them had no formal education. The most frequent type of occupation was farmer, followed by entrepreneur, housewife, civil servant and private employee. A vast majority (96.5%) of the respondents who willing to pay were married and approximately 44% of the them were living under the poverty line, i.e. <1 million IDR (equivalent to US$74.1). Overall, 52.1% of them had good attitude towards vaccination and almost 70% had poor knowledge about transmission and prevention of EVD.

### WTP for an Ebola vaccine

**Figure 1.**
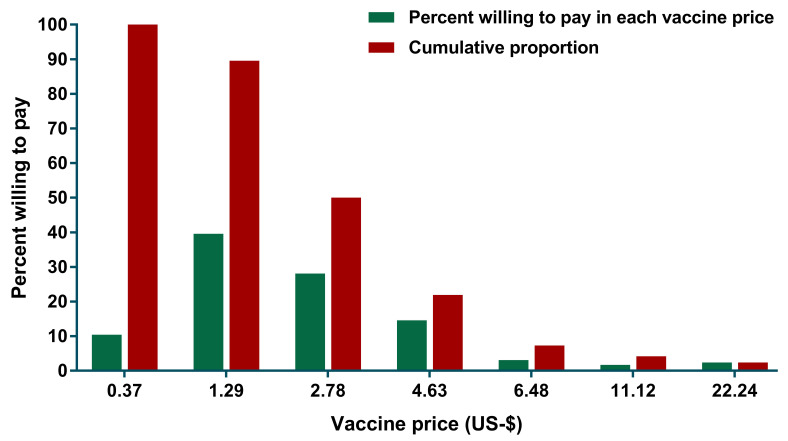
Relationship between the presented vaccine price and proportion of participants who were willing to pay for an Ebola vaccine in Aceh, Indonesia. Only those who were willing to pay for Ebola vaccine were included in this analysis.

Among 288 participants who were willing to pay for Ebola vaccine, 114 (39.6%) of them expressed their WTP at US$ 1.29 and this decreased to 28.1%, 14.6% and 3.1% as the price for Ebola vaccine increased to US$2.78, US$4.63, and US$6.48, respectively ( Figure 1). Only 7 out of 288 respondents agreed to pay the highest price (US$22.24). The mean of WTP was US$2.08 (95% CI: 1.75-2.42).

### Factors associated with WTP for an Ebola vaccine

The initial multivariable model showed that age, educational attainment, type of occupation, marital status, type of residence and having good knowledge of Ebola were associated with WTP with a P-value under 0.25. ( Table 1). The final multivariable model indicated that age, educational attainment, type of occupation, marital status and urbanicity were significantly associated with WTP ( Table 2). Knowledge of Ebola had no association with WTP. Compared to the youngest age group (17–29-year-olds), participants who were between 30–44 years old and those older than 45 years had lower WTP, at approximately US$ 1. Respondents who finished senior high school (year 12) and graduated from university had higher WTP – approximately US$1.7 and US$2.3, respectively, compared to those who had an education less than junior high school (year 9). Compared to farmers, participants who were working as employees in private companies, entrepreneurs and civil servants were willing to pay US$2.6, US$1.8 and US$2.3 more, respectively. In addition, this study found that participants who were married and those who were living in the city had lower WTP compared to unmarried participants and those who were living in the suburbs ( Table 2).

**Table 2.** Final model of factors associated with and willingness-to-pay for a hypothetical Ebola vaccine (N=288).

Parameter	N (%)	Unstandardized coefficients				US$ estimate			*P*-value
		B	95% CI of B		SE	Mean	95% CI		
			Lower	Upper			Lower	Upper	
Intercept		1.478	0.908	2.048	0.290	5.944	4.408	7.479	<0.001
Age group (years) *(Ref: 17–29)*									
30–44	147 (51.0)	-0.311	-0.546	-0.077	0.119	0.993	-0.543	2.528	0.009
≥45	73 (25.3)	-0.299	-0.580	-0.019	0.142	1.005	-0.531	2.540	0.036
Education *(Ref: Less than junior* *high school)*									
Senior high school	136 (47.2)	0.245	0.031	0.458	0.109	1.731	0.195	3.266	0.025
Graduated	40 (13.9)	0.544	0.191	0.897	0.179	2.335	0.799	3.870	0.003
Occupation *(Ref: Farmer)*									
Private employee	24 (8.3)	0.661	0.285	1.037	0.191	2.625	1.090	4.161	0.001
Entrepreneur	72 (25.0)	0.319	0.085	0.552	0.119	1.864	0.329	3.400	0.008
Civil servant	48 (16.7)	0.563	0.225	0.901	0.172	2.379	0.844	3.914	0.001
Marital status *(Ref: Unmarried)*									
Married	278 (96.5)	-0.868	-1.398	-0.337	0.269	0.569	-0.966	2.105	0.001
Urbanicity *(Ref: Suburb)*									
City	41 (14.2)	-0.552	-0.882	-0.221	0.168	0.781	-0.755	2.316	0.001
Knowledge of Ebola *(Ref: Poor)*									
Good	92 (31.9)	-0.206	-0.421	0.008	0.109	1.103	-0.433	2.638	0.059
Mean squared error		0.608							
F value		7.417 (P<0.001)							
R ^2^		0.211							

CI, confidence interval; IDR, Indonesia rupiah; US$, American dollar; SE, standard error; *Ref*, reference group.

## Discussion

WTP is a commonly used method in economic evaluation of healthcare interventions. In cost-benefit analysis, the WTP is able to value both the indirect and intangible aspects of the disease. It is important to be aware of methodology and interpretation of results because of affecting to decision of policy maker and also affecting in national expanded program in immunization on adding new good vaccines ^33^. Several studies have been conducted to assess the WTP for various vaccines and its associated determinants ^34– 36^. One of the reasons since is able to inform future health policies including the adoption of a dengue vaccine

This study was conducted to assess the WTP for a hypothetical Ebola vaccine and its associated determinants among community members in Aceh province, Indonesia. We found that age, educational attainment, type of occupation, marital status and urbanicity were all associated with WTP. Better understanding of which groups have greater WTP for the vaccine and what this amount would be are important to consider if the vaccine were to be introduced into Indonesia in the future.

Age was related to WTP, with younger participants having higher WTP compared to older participants. This corresponds to another study in Indonesia that also showed older participants had lower WTP for a vaccine compared to their younger counterparts ^20^. In Indonesia, this association could arise for several reasons. First, in general, the older generation tends to have lower education levels. In the context of health-related knowledge and WTP, it has been shown that higher education was associated with better health-related knowledge ^37, 38^ and WTP for vaccines against infectious diseases ^39, 40^ although some studies found educational attainment had no association or had no consistent association with WTP ^20, 24, 41, 42^. Second, older community members may have had less exposure to information regarding Ebola, resulting in lower knowledge and awareness of the disease. In addition, many older people work as farmers, have less income and therefore are less willing to pay for vaccination. This could also explain why participants who were working in other sectors had higher WTP compared to farmers.

We also found that participants with higher educational attainment had higher WTP. Higher education level has a positive association with higher WTP in interventions related to infectious diseases ^39, 40^. And it could be that education relates to WTP because of knowledge related to Ebola. However, we found no relationship between knowledge and WTP. It is interesting to discuss why knowledge on Ebola was not significantly associated with WTP in this present study, but higher education was. It could due to the observation that only knowledge on an infectious disease provided health professional was found to be associated with WTP for vaccine ^43^. This is understandable since Ebola, as the new re-emerging infectious disease, was not taught in Indonesia’s curriculum. However, education does increase people’s awareness of infectious diseases and vaccination in general ^44, 45^. Therefore, even though people do not have much knowledge of Ebola, they still have better awareness of the importance to keep themselves protected from infectious diseases, resulting in higher WTP for vaccination as found in this study. Therefore, it is important for the government to target groups with lower education levels during vaccination campaigns to raise their awareness of a specific disease.

Our study found there was no association between income or SES and WTP. However, previous studies have consistently found that income or economic status is one of the most robust predictors for WTP ^20, 25, 39, 40, 42, 46^, i.e., individuals with a higher income can afford a more expensive vaccine. However, one previous study found that income could had negative association with WTP in Nigeria ^47^. The diversity of these findings may serve as an indication that socioeconomic variables behave differently across countries. We note that the vaccine prices that were provided in this present study were substantially lower than the WTP of the respondents. Nevertheless, few respondents (less than 3%) were willing to pay for the vaccine at the highest price (US$22.24) indicating that the provided vaccine prices were not significantly lower than participants’ WTP. According to the theory of goods classification in microeconomics, a negative relationship between income and WTP label the products as inferior goods ^48^. This happen when the consumers have low knowledge and awareness of that particular product and leads the low WTP even though very important.

There are some limitations of this study that need to be discussed. There were no Ebola cases reported in Indonesia and therefore the respondents were provided with brief information related to Ebola infection prior to assess their WTP. Social desirability bias is inevitable in which participants might tend to give favorable answer in some questions given included in WTP section. This study did not explore the WTP difference between two or more scenarios of vaccine with different efficacies. As could be seen in previous studies ^49, 50^, higher efficacy of vaccines resulted in higher value of WTP. This study also did not explore the effect of health insurance on WTP as previous study found that having health insurance were associated with WTP for vaccine ^51^. Finally, a hypothetical bias might exist in which respondents misstate their actual WTP as this study was conducted when no Ebola vaccine had been approved and licensed.

## Conclusions

In this study, the mean of WTP for a hypothetical Ebola vaccine was US$2.08 (95% CI: 1.75-2.42) and the proportion of respondents who were willing to pay for the vaccine decreased with increase of vaccine price. Younger and unmarried participants, those with higher educational attainment and those who were living in the suburbs had higher WTP. In addition, compared to farmers, private employee, entrepreneurs and civil servants also had higher WTP. Better understanding which groups are more willing to pay for the vaccine and what this amount are important to consider if the vaccine were to be introduced into Indonesia in the future.

## Data availability

### Underlying data

Figshare: Willingness-to-pay for a hypothetical Ebola vaccine in Indonesia: A cross-sectional study in Aceh. https://doi.org/10.6084/m9.figshare.9256037 ^32^.

This project contains answers given to each question by each participant.

### Extended data

Figshare: Willingness-to-pay for a hypothetical Ebola vaccine in Indonesia: A cross-sectional study in Aceh (Questionnaire). https://doi.org/10.6084/m9.figshare.9293378.v1 ^21^.

This project contains the questionnaire in Indonesian (original) and English.

Data are available under the terms of the Creative Commons Attribution 4.0 International license (CC-BY 4.0).
