# Supplementary material for: Willingness-to-pay for a hypothetical Ebola vaccine in Indonesia: A cross-sectional study in Aceh
Source: F1000Res. 2023 Jun 14;8:1441. Originally published 2019 Aug 15. [Version 3] doi: 10.12688/f1000research.20144.3 (PMC7194337; doi:10.12688/f1000research.20144.3)
Supplement: Peer review history

# Peer review: Reviewer response for version 3

**DOI:** 10.5256/f1000research.151443.r178680

**Published:** 2023-06-29

**Authors:** Jing-Xin Li

**Affiliations:** 1 Vaccine Clinical Evaluation Department, Jiangsu Provincial Center for Disease Control and Prevention, Nanjing, China

In general, this manuscript is well-written and interesting which can provide evidence for Indonesia’s policies on Ebola vaccines. However, there are several minor points that need to clarify.

1. In introduction, the authors can introduce Ebola vaccines approved for use and their prices. Why did you use a hypothetical vaccine?
2. In “study design and setting”, “The study was conducted in.” The sentence is not complete.
3. How to calculate the minimum sample size?
4. “To recruit the samples, four regencies were selected randomly, and both urban and suburban areas were included.” While in abstract the authors said five cities.
5. In “data analysis”, please clarify “three assumptions of linear regression”.
6. All explanatory variables that had P>0.25 in this model were excluded from final linear regression model 2. Why did the authors choose 0.25 instead of 0.05?
7. About Marital status: does the difference of the sample size between unmarried and married participants influence the conclusion, urbanicity as well? Please provide power value or discuss in limitation briefly.

Is the work clearly and accurately presented and does it cite the current literature?

Partly

If applicable, is the statistical analysis and its interpretation appropriate?

Partly

Are all the source data underlying the results available to ensure full reproducibility?

Yes

Is the study design appropriate and is the work technically sound?

Partly

Are the conclusions drawn adequately supported by the results?

Partly

Are sufficient details of methods and analysis provided to allow replication by others?

Yes

Reviewer Expertise:

Vaccine Clinical Evaluation

I confirm that I have read this submission and believe that I have an appropriate level of expertise to confirm that it is of an acceptable scientific standard.

# Peer review: Reviewer response for version 2

**DOI:** 10.5256/f1000research.27708.r67026

**Published:** 2020-07-15

**Authors:** Brian Godman

**Affiliations:** 1 Pharmacoepidemiology and Pharmacy Practice, Strathclyde Institute of Pharmacy and Biomedical Science, University of Strathclyde, Glasgow, UK

Thank you for the revisions - now happy with the revised paper.

Is the work clearly and accurately presented and does it cite the current literature?

No

If applicable, is the statistical analysis and its interpretation appropriate?

Yes

Are all the source data underlying the results available to ensure full reproducibility?

No

Is the study design appropriate and is the work technically sound?

Yes

Are the conclusions drawn adequately supported by the results?

Yes

Are sufficient details of methods and analysis provided to allow replication by others?

Yes

Reviewer Expertise:

My areas of interest including enhancing the quality and efficiency of the use of medicines across countries including LMICs. This includes WTP for medicines including vaccines. I have published to date over 250 papers - many of which are listed in PubMed

I confirm that I have read this submission and believe that I have an appropriate level of expertise to confirm that it is of an acceptable scientific standard.

# Peer review: Reviewer response for version 2

**DOI:** 10.5256/f1000research.27708.r67025

**Published:** 2020-07-13

**Authors:** Trung Quang Vo

**Affiliations:** 1 Department of Economic and Administrative Pharmacy, Faculty of Pharmacy, Pham Ngoc Thach University of Medicine, Ho Chi Minh City, Vietnam

Is the work clearly and accurately presented and does it cite the current literature?

Partly

If applicable, is the statistical analysis and its interpretation appropriate?

Yes

Are all the source data underlying the results available to ensure full reproducibility?

Yes

Is the study design appropriate and is the work technically sound?

Yes

Are the conclusions drawn adequately supported by the results?

Yes

Are sufficient details of methods and analysis provided to allow replication by others?

Yes

Reviewer Expertise:

Health economic, Public health and Pharmacy Administration

I confirm that I have read this submission and believe that I have an appropriate level of expertise to confirm that it is of an acceptable scientific standard.

# Peer review: Reviewer response for version 1

**DOI:** 10.5256/f1000research.22124.r62295

**Published:** 2020-05-14

**Authors:** Brian Godman

**Affiliations:** 1 Pharmacoepidemiology and Pharmacy Practice, Strathclyde Institute of Pharmacy and Biomedical Science, University of Strathclyde, Glasgow, UK

Thank you for submitting this interesting paper. However, I am not sure regarding the rationale - although you have highlighted this at the beginning of the paper - but even more unlikely currently with travel bans, etc., as a result of COVID-19. This is because I believe Ebola is currently confined to the Congo (and hopefully now reducing) with ongoing steps in the neighbouring countries to prevent the infection spreading. I am not sure therefore of the hypothetical situation regarding Ebola in Indonesia - so good to discuss this more to put the findings into context.

 This is because we have seen similar WTP approaches in Brazil for hypothetical and potential vaccines in key infectious disease areas (different to the situation in Indonesia currently with Ebola), with the need to balance the availability of the vaccine against other protective measures - so good to expand on this in the Introduction. Refs include: (i) Godoi IP et al. Consumer Willingness to Pay for Dengue Vaccine (CYD-TDV, Dengvaxia(R)) in Brazil; Implications for Future Pricing Considerations ^1^; Muniz Júnior RL et al. Consumer willingness to pay for a hypothetical Zika vaccine in Brazil and the implications. Expert review of pharmacoeconomics & outcomes research ^2^ and Sarmento TTR et al. Consumer willingness to pay for a hypothetical chikungunya vaccine in Brazil and the implications. Expert review of pharmacoeconomics & outcomes research ^3^. This builds also on studies discussing the economic impact, etc., of infectious diseases such as dengue - Godoi IP et al. Economic and epidemiological impact of dengue illness over 16 years from a public health system perspective in Brazil to inform future health policies including the adoption of a dengue vaccine. Expert review of vaccines ^4^.

 In addition - good to compare the findings and differences in WTP between different groups for the Ebola vaccine in Indonesia and Brazil as both middle income countries. This can include difference in WTP for a vaccine and any potential rationale. This is very different to just concentrating on e.g. US nationals in West Africa - with very different income levels in Brazil - more akin to Indonesia.

Is the work clearly and accurately presented and does it cite the current literature?

No

If applicable, is the statistical analysis and its interpretation appropriate?

Yes

Are all the source data underlying the results available to ensure full reproducibility?

No

Is the study design appropriate and is the work technically sound?

Yes

Are the conclusions drawn adequately supported by the results?

Yes

Are sufficient details of methods and analysis provided to allow replication by others?

Yes

Reviewer Expertise:

My areas of interest including enhancing the quality and efficiency of the use of medicines across countries including LMICs. This includes WTP for medicines including vaccines. I have published to date over 250 papers - many of which are listed in PubMed

I confirm that I have read this submission and believe that I have an appropriate level of expertise to confirm that it is of an acceptable scientific standard, however I have significant reservations, as outlined above.

## References

[1] : Consumer Willingness to Pay for Dengue Vaccine (CYD-TDV, Dengvaxia®) in Brazil; Implications for Future Pricing Considerations. *Front Pharmacol* .2017;8: 10.3389/fphar.2017.00041 41 10.3389/fphar.2017.00041 28210223PMC5288336

[2] : Consumer willingness to pay for a hypothetical Zika vaccine in Brazil and the implications. *Expert Rev Pharmacoecon Outcomes Res* .2019;19(4) : 10.1080/14737167.2019.1552136 473-482 10.1080/14737167.2019.1552136 30468095

[3] : Consumer willingness to pay for a hypothetical chikungunya vaccine in Brazil and the implications. *Expert Rev Pharmacoecon Outcomes Res* .2019; 10.1080/14737167.2020.1703181 1-8 10.1080/14737167.2020.1703181 31815561

[4] : Economic and epidemiological impact of dengue illness over 16 years from a public health system perspective in Brazil to inform future health policies including the adoption of a dengue vaccine. *Expert Rev Vaccines* .17(12) : 10.1080/14760584.2018.1546581 1123-1133 10.1080/14760584.2018.1546581 30417706

# Peer review: Reviewer response for version 1

**DOI:** 10.5256/f1000research.22124.r62583

**Published:** 2020-04-30

**Authors:** Trung Quang Vo

**Affiliations:** 1 Department of Economic and Administrative Pharmacy, Faculty of Pharmacy, Pham Ngoc Thach University of Medicine, Ho Chi Minh City, Vietnam

Firstly, I give you a big praise for all the efforts you made in the paper. This is a well-designed study with appropriate methods and explicit results so this paper should be accepted. However, there are minor points that should be clarify to improve your paper.

 The following are my comments on your manuscript:

1. The manuscript needs the use of a language editing service. Mistakes in grammar were made, in some cases, even the meaning got lost.
2. The Introduction should be strengthened. I did not see the importance of getting vaccine against Ebola in this part.
3. Although the aim of the study was to “investigate community WTP of Ebola”, only patients’ family members were recruited. I think the study subjects did not consistent with its objective. Perhaps, you should consider to change the term “community WTP” into more suitable one.
4. I know you used contingent valuation method to investigate the WTP even though no information provided. I think you should name the method and the technique used for a better understanding.
5. Please give more explanation: how did you set the range of vaccine price?
6. Please give more explanation: why did you collapse participant into three groups of age? Did they have any significance?
7. I do not think you should define all the variables in the section “Explanatory variables”. Readers can find the groups of each variable in the Table. Please be shortened.
8. Please give more explanation: why did you set the cut-off point of 75% to divide attitude and knowledge into “good” and “poor”?
9. Please cite the reference for this information: “all explanatory variables that had P > 0.25 in this model were excluded from final linear regression model.
10. You said that you had provided information to the Ebola disease prior to assess WTP. I wonder if this step would affect questions regarding the knowledge about Ebola, therefore affect the WTP. Please consider carefully if you still want to state this step on the paper.
11. One of your finding is that higher education associated with higher WTP but the knowledge did not. I think you should give more discussion on this interesting finding.
12. In discussion section, you should state more about benefits of WTP assessment on the economic evaluation. Please add this statement on your discussion: “…WTP is a commonly used method in economic evaluation pf healthcare interventions. In cost-benefit analysis, the WTP method can value both the indirect and intangible aspects of a disease or condition. In cost-effectiveness or cost-utility analysis, WTP is consider to be a reference value to assess if an intervention is cost-effective or not ^1^. It is important to be aware of methodology and interpretation of results because of affecting to decision of policy maker and also affecting in national expanded program in immunization on adding new good vaccines ^2^.”
13. In limitation, you should state that this study did not explore the effects of vaccine efficacy on WTP values. Please add this statement under limitation: “This study did not explore the WTP difference between two or more scenarios of vaccine with different efficacy. As could be seen in previous study, higher efficacy of vaccines resulted in higher value of WTP ^3,4,5^”

Is the work clearly and accurately presented and does it cite the current literature?

Partly

If applicable, is the statistical analysis and its interpretation appropriate?

Yes

Are all the source data underlying the results available to ensure full reproducibility?

Yes

Is the study design appropriate and is the work technically sound?

Yes

Are the conclusions drawn adequately supported by the results?

Yes

Are sufficient details of methods and analysis provided to allow replication by others?

Yes

Reviewer Expertise:

Health economic, Public health and Pharmacy Administration

I confirm that I have read this submission and believe that I have an appropriate level of expertise to confirm that it is of an acceptable scientific standard.

## References

[1] : Essentials of Pharmacoeconomics. *Lippincott Williams and Wilkins* .2013;

[2] : The Economic Evaluation in Vaccines - A Systematic Review in Vietnam Situation. *Systematic Reviews in Pharmacy* .2018;9(1) : 10.5530/srp.2018.1.1 1-5 10.5530/srp.2018.1.1

[3] : Customers' preferences and willingness to pay for a future dengue vaccination: a study of the empirical evidence in Vietnam. *Patient Prefer Adherence* .2018;12: 10.2147/PPA.S188581 2507-2515 10.2147/PPA.S188581 30568429PMC6267625

[4] : A Multi-country Study of the Household Willingness-to-Pay for Dengue Vaccines: Household Surveys in Vietnam, Thailand, and Colombia. *PLoS Negl Trop Dis* .2015;9(6) : 10.1371/journal.pntd.0003810 e0003810 10.1371/journal.pntd.0003810 26030922PMC4452082

[5] : Acceptability of hypothetical dengue vaccines among travelers. *J Travel Med* .20(6) : 10.1111/jtm.12056 346-51 10.1111/jtm.12056 24165380

# Peer review: Reviewer response for version 1

**DOI:** 10.5256/f1000research.22124.r61990

**Published:** 2020-04-21

**Authors:** Jing-Xin Li

**Affiliations:** 1 Vaccine Clinical Evaluation Department, Jiangsu Provincial Center for Disease Control and Prevention, Nanjing, China

For novel vaccines (e.g., Ebola vaccines), WTP was often considered as a major factor of vaccine policy and innovation ^1^, ^2^. Several studies have examined WTP for an Ebola vaccine in Ebola-affected countries (e.g., West Africa ^3^, ^4^, ^5^ and America ^6^. This study in Indonesia helped to supplement and enrich WTP for an Ebola vaccine in non-Ebola- affected countries.

 However, there are several significant omissions in study design from this manuscript. 

1. Although this study aimed to investigate community WTP for Ebola vaccine in Indonesia, the selection of respondents (patients’ family members who visited outpatient departments) caused selection bias.
2. This study was conducted in Aceh province, Indonesia. Aceh comprises predominantly rural areas ^7^, which might result in the skewed distribution of participants’ characteristics, including Education (Senior high school: 47.2%), Occupation (Farmer: 26.7%) and (Suburb: 85.8%). As a result, some conclusions that high educational attainment, working as a private employee, entrepreneur or civil servant (compared to farmers) and residing in a suburb (compared to a city) were associated with higher WTP could not exclude the influence of these skewness distributions.
3. The authors stated that, “Prior to assessing their WTP, participants were provided with an introduction to the Ebola disease including the symptoms and modes of transmission.” Although there were no Ebola cases reported in Indonesia, some respondents were still likely to learn about Ebola and Ebola vaccines in a variety of ways, such as the Internet or newspapers. In addition, knowledge of vaccines had proven to be one of the crucial variables affecting WTP ^7^. Therefore, this intervention would bias the outcome of WTP, which did not reflect the real WTP of respondents.

Is the work clearly and accurately presented and does it cite the current literature?

Partly

If applicable, is the statistical analysis and its interpretation appropriate?

Partly

Are all the source data underlying the results available to ensure full reproducibility?

Yes

Is the study design appropriate and is the work technically sound?

Partly

Are the conclusions drawn adequately supported by the results?

Partly

Are sufficient details of methods and analysis provided to allow replication by others?

Yes

Reviewer Expertise:

Vaccine Clinical Evaluation

I confirm that I have read this submission and believe that I have an appropriate level of expertise to state that I do not consider it to be of an acceptable scientific standard, for reasons outlined above.

## References

[1] : Dengue vaccine acceptance and willingness to pay. *Human Vaccines & Immunotherapeutics* .2017;13(4) : 10.1080/21645515.2016.1259045 786-790 10.1080/21645515.2016.1259045 PMC540436927905832

[2] : Policy making for vaccine use as a driver of vaccine innovation and development in the developed world. *Vaccine* .2017;35(10) : 10.1016/j.vaccine.2016.10.080 1380-1389 10.1016/j.vaccine.2016.10.080 28253995

[3] : Acceptability and Willingness-to-Pay for a Hypothetical Ebola Virus Vaccine in Nigeria. *PLoS Negl Trop Dis* .2015;9(6) : 10.1371/journal.pntd.0003838 e0003838 10.1371/journal.pntd.0003838 26076007PMC4467844

[4] : Knowledge and attitudes about Ebola vaccine among the general population in Sierra Leone. *Vaccine* .2016;34(15) : 10.1016/j.vaccine.2016.02.046 1767-72 10.1016/j.vaccine.2016.02.046 26928073

[5] : Health-Care Workers' Perspectives on Ebola Virus Vaccine: A Focus Group and In-Depth Interview Interventional Study. *The American Journal of Tropical Medicine and Hygiene* .2016;95(3) : 10.4269/ajtmh.16-0206 654-662 10.4269/ajtmh.16-0206 27382077PMC5014274

[6] : Willingness to pay for an Ebola vaccine during the 2014–2016 ebola outbreak in West Africa: Results from a U.S. National sample. *Human Vaccines & Immunotherapeutics* .2018;14(7) : 10.1080/21645515.2018.1423928 1665-1671 10.1080/21645515.2018.1423928 PMC606787629333950

[7] : Willingness to pay for a dengue vaccine and its associated determinants in Indonesia: A community-based, cross-sectional survey in Aceh. *Acta Trop* .2017;166: 10.1016/j.actatropica.2016.11.035 249-256 10.1016/j.actatropica.2016.11.035 27908746

# Peer review: Reviewer response for version 1

**DOI:** 10.5256/f1000research.22124.r52546

**Published:** 2019-08-28

**Authors:** Roger Chun-Man Ho

**Affiliations:** 1 Department of Psychological Medicine, Yong Loo Lin School of Medicine, Biomedical Global Institute of Healthcare Research & Technology (BIGHEART), National University of Singapore, Singapore, Singapore

Thank you for inviting me to reviewing the paper “Willingness-to-pay for a hypothetical Ebola vaccine in Indonesia: A cross-sectional study in Ace”. This is an important and well written paper with sound methodology. I have the following recommendations to improve this paper.

1. Under discussion, the authors stated that, “However, we found no relationship between knowledge and WTP. It is interesting to discuss why knowledge on Ebola was not significantly associated with WTP in this present study, but higher education was.” The authors should state that knowledge on an infectious disease provided health professional was found to be associated with WTP. Please add the following statement:
  …. but higher education was. It could due to the observation that only knowledge on an infectious disease provided health professional was found to be associated with WTP for vaccine. ^1^
2. Under limitation, the authors should state that this study did not explore on the effect of insurance on WTP. Please add the following statement under limitation:
  ….. some questions given included in WTP section. This study did not explore the effect of health insurance on WTP as previous study found that having health insurance were associated with WTP for vaccine. ^2^

Is the work clearly and accurately presented and does it cite the current literature?

Partly

If applicable, is the statistical analysis and its interpretation appropriate?

Yes

Are all the source data underlying the results available to ensure full reproducibility?

Yes

Is the study design appropriate and is the work technically sound?

Yes

Are the conclusions drawn adequately supported by the results?

Yes

Are sufficient details of methods and analysis provided to allow replication by others?

Yes

Reviewer Expertise:

Willingness to pay on vaccine

I confirm that I have read this submission and believe that I have an appropriate level of expertise to confirm that it is of an acceptable scientific standard.

## References

[1] : Knowledge, attitude, and practice on and willingness to pay for human papillomavirus vaccine: a cross-sectional study in Hanoi, Vietnam. *Patient Prefer Adherence* .2018;12: 10.2147/PPA.S165357 945-954 10.2147/PPA.S165357 29881260PMC5985800

[2] : Feasibility and willingness to pay for dengue vaccine in the threat of dengue fever outbreaks in Vietnam. *Patient Prefer Adherence* .2018;12: 10.2147/PPA.S178444 1917-1926 10.2147/PPA.S178444 30288032PMC6163003
